# Novel nicotine products: Averting a harmful revolution

**DOI:** 10.18332/tid/172415

**Published:** 2023-10-24

**Authors:** Laura Jovell, Adrián González-Marrón, Cristina Lidón-Moyano, Jose M. Martínez-Sánchez

**Affiliations:** 1 Group of Evaluation of Health Determinants and Health Policies, Faculty of Medicine and Health Science, Universitat Internacional de Catalunya, Barcelona, Spain

**Keywords:** novel nicotine products, policies, advertising

**Dear Editor,**

The implementation of policies to regulate tobacco advertising is one of the most cost-effective measures to fight against smoking and the nicotine consumption epidemic. As outlined in Article 13 of the World Health Organization Framework Convention on Tobacco Control^1^, these policies should spread over all forms of commercial communication that directly or indirectly promote any novel nicotine product or its use.

However, for over a decade, the use of novel nicotine products has spread around the world, including products such as electronic nicotine delivery systems (ENDS), heated tobacco products (HTPs), and nicotine pouches, among others. The recent irruption of these products into the market sector exposed a loophole in the legislation. The tobacco industry has taken advantage of it by advertising and promoting these new forms of nicotine consumption like any other consumer product. Some examples can be found as explicit advertising or as product placement in films, television, social media, and sports sponsorships^2,3^. This loophole exploitation is one of the main factors that has contributed to the popularity and increased consumption of these novel nicotine products in recent years, especially in the younger population^4,5^.

The situation escalated reaching such a point that four years ago the use of ENDS among youth was declared an epidemic in the USA^6^. Although some laws exist^7^, in some cities of Europe we could still find ENDS and HTP advertising in public areas, such as those found in London this spring or Girona (Catalonia) right before the pandemic, besides non-compliance with the anti-smoking regulation (Figures 1A and 1B). Furthermore, the tradition continues as shops selling novel tobacco products sometimes have point-of-sale promotion displaying unregulated full-sized advertisements in the front window, as found in Vienna this summer (Figure 1D) and in Florence in 2022 (Figure 1C), or digital advertising that can still be found on the Internet as of today (Figure 1D).

**Figure 1:**
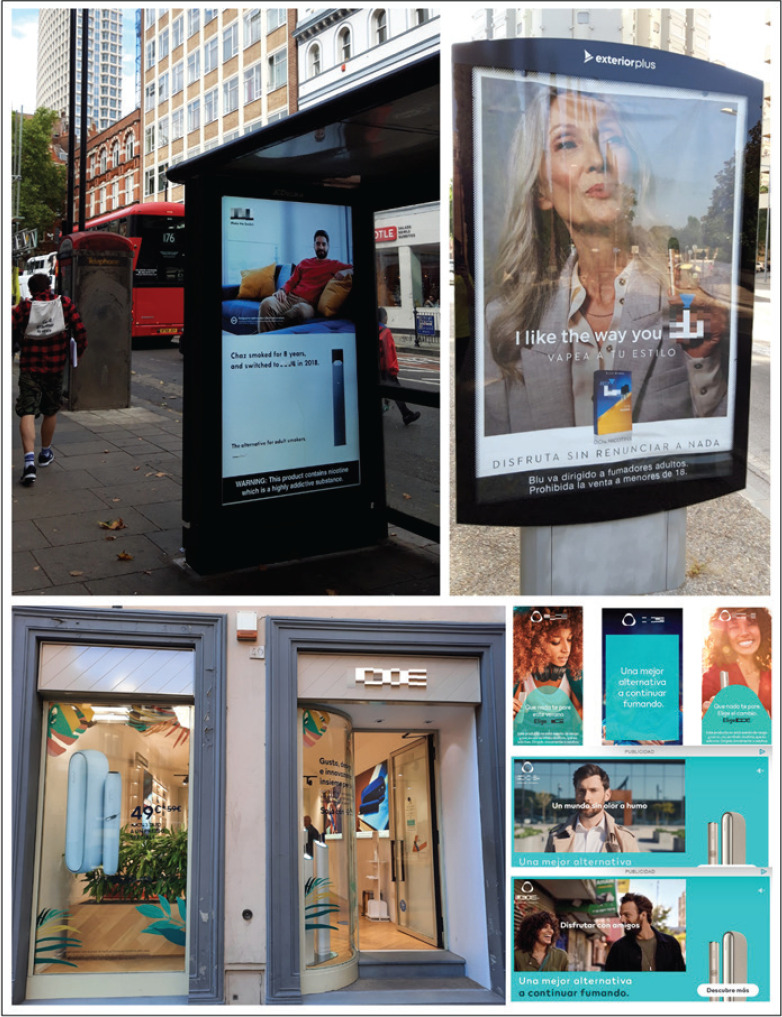
A) Advertisement in Gray’s Inn Road near King’s Cross Station in London, UK. B) Advertisement a few meters away from the city’s courthouse entrance, the Palau de Justícia in Girona, Catalonia, Spain. C) Advertising for HTP in the front window of a shop in Via della Vigna Nuova, a street next to Via de’ Tornabuoni, a luxurious shopping street in the historic center of Florence, Italy. D) Screen captures of digital advertising in Spanish of HTP in an entertainment online magazine and digital national newspaper

Advertisements remain a trigger that favors the consumption of novel nicotine products, which can have lasting harmful effects on health^8^, especially when exposed from a very early age^9^. Instead of decreasing the prospects of smoking conventional tobacco, the use of novel nicotine products seems to increase its likelihood, particularly among youth^10^. This reflects the importance of public health surveillance and the implementation of prevention and control programs. Measures such as limiting where and how they are advertised and sold could help mitigate the effects on today’s youth, preventing consumption in future generations. Despite the emerging new policies regulating novel nicotine products in Europe, Canada, and the USA, most existing strategies targeted conventional tobacco. Nonetheless, recently new advances are being made to properly regulate them in some EU member states^5^, a trend others should follow.

Explicit and up-to-date advertising regulations of novel nicotine products, adapting to anticipate the commercial interests of tobacco companies, are necessary in view of the current passivity and waywardness, ensuring thereafter compliance with the law. The weight of this responsibility falls not only on the governments to establish laws, but on communities and cities to see that regulations are properly implemented.

## Data Availability

The photos included in this article were taken by the authors. No additional permission is required to use these images in this article.

## References

[1] WHO Framework Convention on Tobacco Control (2013). Guidelines for implementation: article 13.

[2] Global Center for Good Governance in Tobacco Control (2020). Why Big Tobacco sponsors Formula 1.

[3] Kostygina G, Tran H, Schillo B, Silver NA, Emery SL (2022). Industry response to strengthened regulations: amount and themes of flavoured electronic cigarette promotion by product vendors and manufacturers on Instagram. Tob Control.

[4] Centers for Disease Control and Prevention (2016). E-cigarette ads reach nearly 7 in 10 middle and high-school students.

[5] Snell LM, Nicksic N, Panteli D (2021). Emerging electronic cigarette policies in European member states, Canada, and the United States. Health Policy.

[6] Office of the US Surgeon General, US Centers for Disease Control and Prevention, Office on Smoking and Health (2018). Surgeon General’s Advisory on E-cigarette Use Among Youth.

[7] European Parliament (2014). Directive 2014/40/EU of the European Parliament and of the Council of 3 April 2014 on the approximation of the laws, regulations and administrative provisions of the Member States concerning the manufacture, presentation and sale of tobacco and related products and repealing Directive 2001/37/EC.

[8] McGrath-Morrow SA, Gorzkowski J, Groner JA (2020). The effects of nicotine on development. Pediatrics.

[9] Hammond D, Reid JL, Rynard VL (2021). Indicators of dependence and efforts to quit vaping and smoking among youth in Canada, England and the USA. Tob Control.

[10] Gallus S, Borroni E, Odone A (2021). The role of novel (tobacco) products on tobacco control in Italy. Int J Environ Res Public Health.

